# A survey to explore the psychological and professional impact of change imposed by COVID-19

**DOI:** 10.2144/fsoa-2023-0057

**Published:** 2023-06-24

**Authors:** Mona Boudabbous, Nada Charfi, Héla Gdoura, Lassad Chtourou, Manel Moalla, Leila Mnif, Ali Amouri, Mohamed Maalej, Nabil Tahri

**Affiliations:** 1 Gastroenterology Departement, Hédi Chaker Hospital, Sfax, Tunisia; 2 Psychiatry C Departement, Hédi Chaker Hospital, Sfax, Tunisia; 3 Medecin B Departement, Medecin Sfax University, Sfax University, Sfax, Tunisia

**Keywords:** change management, comprehension, COVID-19, information, participation, readiness, resistance

## Abstract

**Aim:**

Staff of a department underwent a change following the COVID-19 pandemic. The objective of this study was to assess the psychological and professional impact of this change.

**Materials & methods:**

This is cross-sectional study, including all department staff conducted from 15 July 2021 to 15 August 2021

**Results:**

All the staff concerned (n = 36) participated. Only 9.37% declared that they understood the change. The main problem encountered by participants was poor communication (86.2%). More than 75% among participants had a demotivation and 54.5% had resisted. The main cause of resistance was quality of organization (42.4%).

**Conclusion:**

Our study illustrates the difficulties encountered by actors involved in organizational change and the high risk of their resistance.

Coronavirus belongs to the family Coronaviridae (subtype Coronavirinae and genus *βetacoronavirus*). It was transmitted to humans probably by pangolin, in a seafood market in Wuhan, China, in December 2019 [1]. The discovery of pneumonia of unknown etiology prompted Chinese researchers to rapidly isolate the pathogen in January 2020. The rapidity of human-to-human transmission caused a pandemic. On 11 February, WHO named the respiratory disease, caused by SARS-CoV-2, COVID-19. It is considered as a public health emergency of international concern. Currently, the most reliable technique for diagnosis of COVID-19 is performed by specific RT-PCR on a nasopharyngeal swab. The result can be obtained usually within 4 h [2].

Up to May 2020, more than 3.3 million confirmed cases of COVID-19 and over 230,000 deaths due to the disease have been reported worldwide [3]. At that time, Tunisia was relatively spared during the first wave (45 deaths in 3 months) because it did not hesitate to institute a 3-month national containment as soon as the first cases appeared in March 2020. This measure could not avoid the consequences of subsequent waves, especially those of the beginning of 2021, corresponding to the third wave and causing an average of 70 deaths per day with a first peak of 103 deaths on 22 January, then a second peak of 119 deaths on 28 April. The maximum peak was observed during the fourth wave that had started in July 2021, with a total of 381 deaths on 1 August [4]. This deterioration in the health situation can be explained by several reasons: the relaxing of barrier measures; the reduction of control procedures at the borders; gatherings during social movements, lack of resources in hospitals; delay of the anti-COVID vaccination (the vaccination campaign having started only on 13 March 2021, whereas that of neighboring countries was started in January and the arrival, in April–May 2021, of the Delta strain, which is more contagious and more virulent than the previous ones [5].

According to WHO recommendations, confirmed patients should be placed in isolation either at home or in a healthcare facility, and hygiene rules should be respected. Their management will depend on the clinical form of the disease. The use of antivirals, immunomodulators, serotherapy, chloroquine or hydroxychloroquine with azithromycin is not recommended outside of clinical trials. Corticosteroid therapy is reserved for severe forms of the disease and systematic antibiotic therapy is not indicated outside of a clinical suspicion or in severely ill patients. In fact, the drug treatment of COVID-19 can be divided schematically into three phases: the first phase is post-exposure prophylaxis, which is the prevention of infection with SARS-CoV-2 after contact with the virus, especially in those at high risk of developing into a severe form. This is then followed by an early phase of infection, asymptomatic or symptomatic, in which patients are taken into care in an ambulatory setting, possibly followed by a later phase in which patients are taken into care in the hospital in a conventional or intensive care unit [6].

The impact of the health crisis due to the COVID-19 pandemic was considerable, worldwide, in terms of collective and individual commitment, adaptation, health, very rapid adoption of new ways of working. Companies were challenged to adjust very quickly to cope with the emergency [7]. In this context, several changes have been imposed, such as for a gastroenterology department whose staff was forced to leave its premises, to work in other narrower and geographically distant ones. Thus, since 2 January 2020, the medical and paramedical staff of this service has undergone a radical and brutal change of premises with a halt to endoscopic activities for a year and a half. The majority of the staff moved with the patients, except for a few people who continued to work in the old premises, providing care to patients infected with COVID-19. However, any change is fraught with challenges: if successful, it leads to an improvement in the state of things, but if unsuccessful, it leads to difficulties and resistance. If the evaluation of the implementation of change has not yet received the attention it deserves, more research on the reactions of employees to change seems necessary [8]. For this reason, we conducted this study to assess the staff’s experience of change and to investigate possible factors causing failures.

## Materials & methods

This was a cross-sectional study conducted as a survey from 15 July 2021 to 15 August 2021 using an electronic questionnaire sent by email. A paper was provided to participants who preferred this method. We included all the personnel assigned to the gastroenterology department during the study period. Any staff having a seniority of exercise lower than 6 months was excluded from this study. The questionnaire was used for the first time because of the recent nature of the disease. Thus, no pilot study to validate the questionnaire was conducted.

After explaining the aims and modalities of the questionnaire, we received oral consent from the participants.

The questions included the epidemiological data (age, sex, profession, length of time working in the department, possible history of a similar situation in other departments), the perception of the change and its psychological and professional consequences, the problems encountered and the proposed solutions.

Computer entry and statistical analysis of the data were performed with the statistical software Statistical Package for Social Sciences (SPSS) for Windows version 21. We expressed categorical variables as frequencies and quantitative variables as means ± standard deviation after checking the normality of the distribution, or as median and interquartile range if the normality of the distribution was not checked. The Kolmogorov–Smirnov test was used to check the normality of the distribution of quantitative variables.

## Results

All relevant staff participated. There were 36 participants, 11 (30.55%) of whom were between 50 and 60 years old and 10 (27.77%) between 40 and 50 years old. A predominance of women was noted with a sex ratio M/F of 0.38. The majority of participants were physicians (41.66%), followed by workers (22.22%). Among the 15 physicians, nine were residents (60%), one university hospital assistant, two associate professors and three professors. The length of service in the gastroenterology department was 10–20 years for ten participants (27.7%) and 2–10 years for nine participants (25%) and 20–30 years for eight participants (22.2%). Of the 36 participants, 16 (44.4%) had already experienced a change of workplace, of which nine (56.25%) thought it was a negative experience. Only 32 (88.8%) participants indicated their level of information about the proposed change prior to its implementation. Prior to the adoption of the change project, participants’ level of information by the project varied: five (15.6%) reported hearing about the project several times, eight (25%) reported hearing about the project sometimes, seven (21.8%) reported hearing about the project only once while 12 (37.1%) had never heard about the project. The leaders of the change project had never presented this project before its implementation for the majority of the participants (n = 30, 83.3%). They had presented it only once for one participant (2.77%), a few times for four participants (11.1%) and several times for one participant (2.77%). Among 32 participants, only three (9.37%) had reported 100% understanding of the goals and modalities of the change, while 11 (34.37%) only partially understood these elements. Of the remaining 18 participants (56.26%), who reported a complete lack of understanding of the project, eight (44.4%) had noted their willingness to understand the objectives and terms of this change while the remaining ten (55.6%) participants had no intention of understanding. Similarly, only two of 29 participants (6.9%) reported having a clear idea of what will change for them, while the majority (n = 17, 58.62%) had no idea. Furthermore, among 31 participants, only one (3.2%) gathered information about the change repeatedly, another participant did so sometimes (3.2%), 11 (35.4%) did not do so but said they wished they had, while 18 (58.2%) had no intention of doing so at all. Similarly, only one participant among 32 (3.125%) had attended several meetings regarding the change, four (12.5%) attended only a few meetings, 16 (50%) could not attend despite their motivation while the remaining 11 (34.375%) were not interested in attending these meetings. Of the 33 participants who answered the question, 28 (84.84%) saw the change as a ‘constraint’, while the other five saw it as an opportunity. In fact, 20 of the 32 participants (62.5%) stated that they did not understand the change and did not embrace it. The main problem encountered by the participants was poor communication (86.2%), followed by lack of clarity of objectives (72.4%) and lack of involvement of managers (72.4%) (Table 1).

**Table 1.** Distribution of participants according to the problems encountered during the change.

Type of problem	n (%)
Poor communication	25 (86.2)
Lack of clarification of objectives	21 (72.4)
Lack of leader’s involvement	21 (72.4)
Poor workload distribution	17 (58.62)

Of 33 participants who responded to the question, 18 (54.5%) reported resistance to change. The main cause of resistance reported by the participants was the quality of the change organization (42.4%) followed by the change strategy (36.3%) (Table 2).

**Table 2.** Distribution of participants according to the causes of their resistance to change.

Type of problem	n (%)
Quality of the change organization	14 (54.5%)
Strategy of the change	12 (42.4%)
Nature of the change	10 (30.3%)
Team working on the change	5 (15.15%)
Individual beliefs	4 (12.12%)
Change’s leader	3 (9.09%)

**Figure 1.**
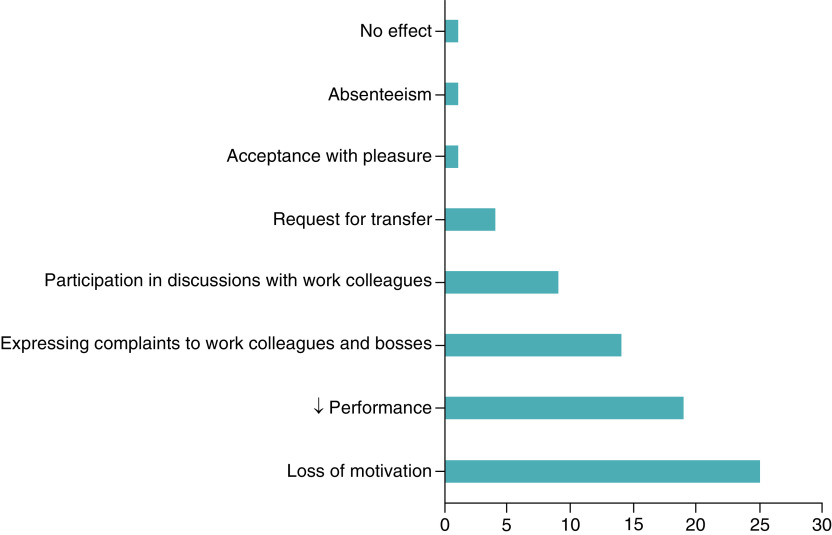
Distribution of participants according to the influence of the change on their behavior at work.

The change influenced participants’ workplace behavior in varying ways (Figure 1). More than 3/4 among the participants (75.75%) experienced demotivation with a feeling of decreased performance in more than half of the participants (n = 19, 57.57%). Of the 33 participants, four requested a job transfer (12.12%).

Of our participants, only 26 specified their feelings toward the agent responsible for the change. Of these, 24 (92.3%) felt hatred or contempt. Of the 33 participants, 31 (94%) were convinced that the change had a negative impact on the functioning of the department. The reasons for the participants’ frustration were dominated by difficult working conditions for the staff who had worked in the n ew premises and the feeling of incompetence toward patients with COVID-19 for the staff designated to manage these patients.

## Discussion

The first cases of the COVID-19 pandemic emerged in Wuhan, China in November 2019. However, vaccination against this disease has encountered a worldwide hesitation. Reasons for vaccine hesitancy against trivial COVID-19 were fear of vaccine side effects, patients felt they were immune to the disease, and preference for other prevention methods [9].

From China, the disease spread around the world, bringing with it a trail of macabre disasters and crises. As in the majority of countries affected by the pandemic, non pharmaceutical measures including physical distancing, proper use of masks, teleworking, isolations and quarantines have been imposed in Tunisia to delay the spread of COVID-19. Moreover, the Tunisian national plan for the fight against COVID-19 provides decided full mobilization of the health system, as well as medical institutions to protect at-risk populations, ensure the care of non serious patients and patients with signs of seriousness in care institutions [10]. However, due to the shortage of emergency departments in our region, some departments, like ours, have been requisitioned by the health authorities and transformed into hospitalization units for infected patients. Then, the staff of our department has experienced a very hard ordeal due to the withdrawal of his usual work premises and the cessation of endoscopic activity.

Our study found that 62.5% of the participants did not understand or embrace change and nearly 85% perceived change as a ‘strain’ inducing demotivation in more than three quarters of them and a sense of decreased performance in more than half of them. While information, the ability to change and participation in change are variables that have already attracted the attention of academics, we share the opinion of other authors who believe that employee understanding is also a major variable that deserves further investigation [8,11]. Indeed, change in and of companies is a crucial area of action and a real managerial challenge for leaders and middle managers in charge of maintaining the competitive vitality of their organization [12]. However, two aspects of organizational change can be distinguished: prescribed change and emergent change. Prescribed change is consciously planned, it is deliberate, it is the product of reasoning and action. It is generally desired by the members of the organization. Emergent change seems to appear spontaneously. In the latter case, change is mainly emergent because of its unpredictability. It appears as a result of the influence of external factors (economic, political, competitive, etc.) or internal factors (power plays, knowledge circulation, uncertainty, etc.) [13]. This change is therefore not initially desired. It is this type of change that has been imposed in our study.

Change implies moving from the known to the unknown. Because the future is uncertain and can have adverse effects on people’s careers, salaries and skills, members of organizations do not generally support change unless there are compelling reasons to do so. This fact explains the lack of motivation of our participants, 63.3% of whom said they had never contacted managers to better understand the change and 58.2% of whom had no intention of gathering information about it at all. In fact, organizational change is generally considered to be associated with the experience of stressful working conditions, uncertainty and anxiety [14]. These situations can both cause stress and have a negative impact on the physical and psychological well-being of employees [15]. As a result, Swanson and Power suggest that managers need to pay close attention to employees’ perceptions during these periods of chronic stress [16]. Among 33 participants, 31 (94%) were confident that the change would have a negative impact on departmental functioning. Similar results have been found in the literature in several studies. For example, in a study conducted by McKinsey, two-thirds of the 3199 business leaders surveyed admitted that the major changes undertaken in recent years had failed to significantly improve the performance of their organization [13]. The same firm had highlighted in 2017 that 70% of change programs ended in failure [17]. In order to explain this lower performance in the conduct of organizational changes, the literature often puts forward the theme of individual resistance. The term resistance to change refers to any behavior or attitude that indicates a refusal to support or make a change to a change project. As illustrated in our study, resistance to change can take many forms. While overt resistance may be expressed in strikes, decreased productivity, poor work quality or even outright sabotage, covert resistance may be expressed in increased tardiness and absenteeism, transfer requests, resignations, loss of motivation, decreased morale and increased accident and error rates [13]. In our study, 54.5% of participants reported resistance to change. The main cause of resistance reported by the participants was the quality of the change organization (42.4%) followed by the change strategy (36.3%). Christophe Peiffer, a human resources consultant, specified the five factors of resistance to change: individual factors, factors related to the nature of the change itself, strategic factors, factors related to the agent of the change and organizational and group factors [18].

With regard to individual factors, each person, because of his or her history, culture, value system or beliefs, has very personal representations of all the elements related to change. For example, an unknown situation may be experienced as a discovery for one person and as a real anxiety for another. In the same way, the preference for stability, the attachment to comfortable habits or the questioning of skills are among the main sources of resistance linked to individual factors. In our study, 44.4% had previously experienced a change of premises, of which 56.25% thought it was a negative experience. This fact could explain in part their refusal to change. Regarding the factors related to the nature of the change itself, it is not the object of the change that is questioned, but the action of changing. Indeed, individuals consider that the time, energy and attention required to carry out a change project are not ‘worth it’. In our study, 55.6% of the participants had no intention of understanding the change prior to its implementation and 58.2% of them had no intention of gathering information about it at all. This result may reflect their belief that the change is unnecessary.

As for the strategic factors, change agents must also be prepared to face resistance related to the strategy implemented to lead the change. In fact, in our study, the quality of the organization of the change was the main cause of resistance according to the participants (54.4%), followed by the strategy of the change (42.4%).

Regarding the resistance factors related to the agent of change, resistance is due to the person who embodies and/or is at the origin of the change project. This factor is more influential if this person does not have close contact with the people involved and seems to have personal interests in the change he or she is proposing, or if he or she is too emotionally involved. Finally, resistance to change may be triggered if the person behind the change has personal characteristics that are very different from those of the people involved in the project (age, education, socio-economic status, etc.). In our study, nearly 60% of the participants were not physicians, yet the leaders of the change (hospital director, president of the medical committee, president of the anti-COVID 19 crisis unit, regional director, etc.) were physicians. This factor could also have contributed to the genesis of the resistance.

Finally, the structure of the organization can be a source of resistance to change. Thus, a bureaucratic organizational structure, such as public hospital structures, generates more resistance than a more flexible structure. While a multitude of change management methodologies exist, their application in complex healthcare contexts remains to be validated. A recent review of the literature published in 2021 included 38 studies reporting the use of 12 change management models in different healthcare settings in ten countries. In this review, the authors identified several change managements models that are being applied to achieve change in healthcare teams, services and organizations [19]. In the articles examined by this review, change management models provide a framework for change agents to consider the key elements necessary for change to occur and be sustained. These key elements include exploring why change is needed and developing the right messages for stakeholders at each stage to accompany them on the change journey. Adopting this strategy would have helped our participants accept and better adapt to change. There are many models for managing organizational change, particularly in the healthcare field. The two most frequently used models are Lewin’s three stages and Kotter’s eight stages [19]. Lewin’s three stages model suggests that practices within an organization are inherently stable and resistant to change. Therefore, change only occurs through the following three stages: the ‘thawing’ stage where those involved are made aware of the need for change; the second stage is represented by ‘change’, in which new systems and behavioral expectations are implemented; and the third stage is ‘refreezing’, in which new ways of thinking and doing are institutionalized and, therefore, solidified into new practices [20]. For Kotter, change has both an emotional and a situational component, and the methods for managing each are expressed in his 8-step model (develop urgency, build a guiding team, create a vision, communicate for buy-in, enable action, create short-term gains, do not let go, and ‘make it stick’) [21]. In all cases, whatever the model advocated, it is crucial to accompany the people concerned in order to avoid their resistance, especially if the change strategy, as in our case, is a hierarchical one where the change is simply announced to the individuals who must follow the recommendations of the managers.

The strength of our study lies in the evaluation of the success of an organizational change through its impact on the actors. We were able to reveal not only the major risk of resistance, the main source of failure, in the absence of effective communication and support upstream, during and downstream of the change, but also the possibility of an improvement in the adaptation of the actors to the change, if the leaders listen to their grievances and proposals. Moreover, Rafferty *et al.* remind us that “*empirical research has shown that high quality communication about change promotes acceptance, openness and involvement in the change process*” [22]. However, our study involved a limited sample, which prevented us from conducting an analytical study in search of factors associated with resistance to change. Furthermore, due to its cross-sectional nature, this study does not allow us to evaluate the evolution of the impact of change on personnel over time. In fact, it has been shown that the evolution of employees’ reaction to change represents a reliable means of evaluating the efforts of organizational change [23].

The main bias of our study is a measurement bias. Indeed, the use of the ‘paper format’ of the questionnaire allowed some participants not to answer certain questions, even though they were ‘mandatory’ in the ‘electronic’ format. We limited this bias by explaining the ‘items’ not understood by the participants through the Tunisian dialect by a single investigator.

## Conclusion

In conclusion, our study shows that organizational change, even if it is radically dictated by ‘difficult’ circumstances and in a short period of time, such as the ‘COVID-19’ pandemic, must obey a methodology based on continuous listening to all the actors in order to facilitate the adoption of change. This data are particularly interesting because change is even a necessity for the institution to grow. In fact, there is ample evidence that flexible and adaptable organizations enjoy a competitive advantage over those who are rigid and static [13]. Further large-scale studies with an analytical study, using a validated questionnaire in the popular dialect, would allow a better assessment of the psychosocial and professional impact of the change. Our study could play the role of a pilot study that highlights the importance of a carefully planned approach that engages all stakeholders in order to achieve a successful professional change, even in a critical situation.

**Summary points**

- More than 75% of the participants were demotivated by the change, with more than half of them (57.57%) feeling a drop in performance.
- Several difficulties were encountered by the participants. They were dominated by difficult working conditions.
- Among the participants, 90.6% experienced the change with a very bad or bad adaptation.
- The majority of participants declared that the main mistake made by the leaders of the change was the lack of involvement of the staff in the planning (77.7%) and in the decision making during the change (50%), followed by the lack of listening to the concerns of the staff (61.1%) and the lack of information (52.7%).
- In healthcare, there are key elements that need to be met in order to make change happen and be sustained. These include exploring the reasons why change is needed and developing clear messages for the different actors.
